# Empirical Determination of Scattering Matrices from Magnetic Molecular Interferometry for Gas–Surface Collisions

**DOI:** 10.1021/acs.jpcc.4c06913

**Published:** 2024-12-02

**Authors:** Helen Chadwick, Gil Alexandrowicz

**Affiliations:** Department of Chemistry, Faculty of Science and Engineering, Swansea University, Swansea SA2 8PP, U.K.

## Abstract

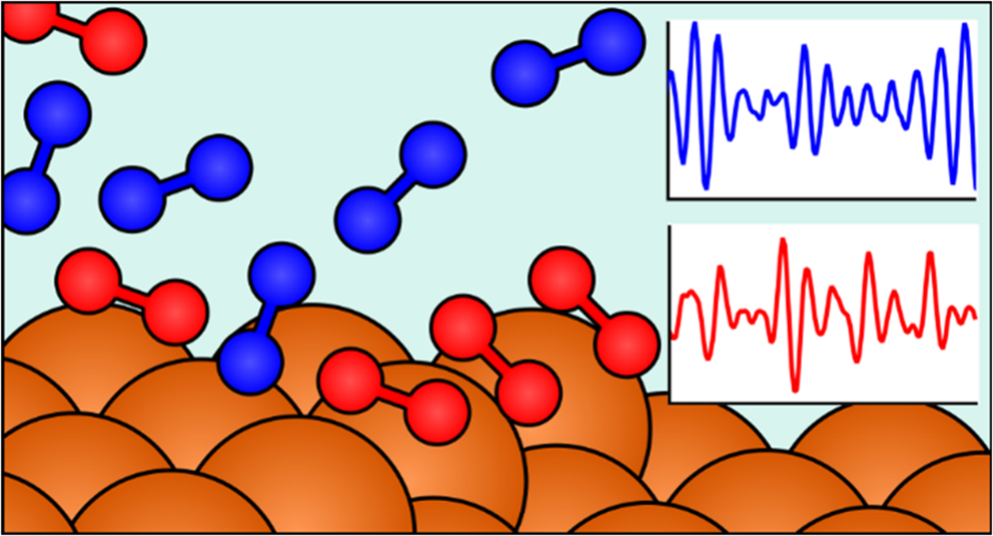

The rotational orientation dependence of H_2_ scattering into different diffraction channels on a Cu(511) surface is studied using a magnetic manipulation interferometry technique. For some channels, markedly different signals are measured, whereas for others, they are more similar. The data are analyzed to obtain scattering matrices, which quantify how the amplitude and phase of the wave function change during the gas–surface collision and are extremely sensitive to the underlying potential. Fits to simulated data with noise at levels comparable to those obtained in the experimental data are also presented, which provide an estimate of the error on the scattering matrix parameters that are obtained, with the uncertainties in the values tending to be lower for the higher order diffraction peaks. The results presented here provide extremely stringent benchmarks for the development of accurate theoretical models, with the number of different channels studied reducing the likelihood of an inaccurate model fortuitously reproducing all the data.

## Introduction

There are many possible outcomes when a molecule collides with a surface from the molecule scattering elastically back into the gas phase with the same energy it started with (i.e., in the same quantum state and translational energy), or it reacting on the surface.^1−3^ It is also possible for inelastic scattering to occur, where the translational, rotational and vibrational energy of the molecule is either exchanged between the molecular degrees of freedom when it collides, or with the surface.^1,4^ A complete picture of the molecule–surface interaction must include all these pathways, the importance of which will change as a function of the molecule’s initial energy and quantum state, as well as the surface material, structure and temperature.

An important concept for describing the interaction between the molecule and the surface and calculating the probabilities of the various possible outcomes of a molecule–surface collision, is a multidimensional potential energy surface (PES), which quantifies how the energy of the system changes as a function of the quantum state of the molecule and its position with respect to the surface. Due to the many degrees of freedom that are involved in describing a molecule–surface collision, it is necessary to make approximations when calculating the PESs. Typically, this involves invoking the Born–Oppenheimer approximation, and then calculating the interaction energy using density functional theory (DFT). Once the system is characterized by a PES, various observables can be calculated including elastic and inelastic scattering probabilities as well as reaction probabilities.^5−10^ Comparing calculated and measured observables yields good agreement in some cases validating the PES and methods used,^11−14^ but in other cases discrepancies are observed,^15−18^ even when the same PES can reproduce other observables for the same system.^19,20^ Different PESs can also reproduce the same experimental observable,^11,12^ which, in principle at least, should not both be correct, suggesting that both more data and/or a different kind of measurement is required to distinguish between the two.

**Figure 1:**
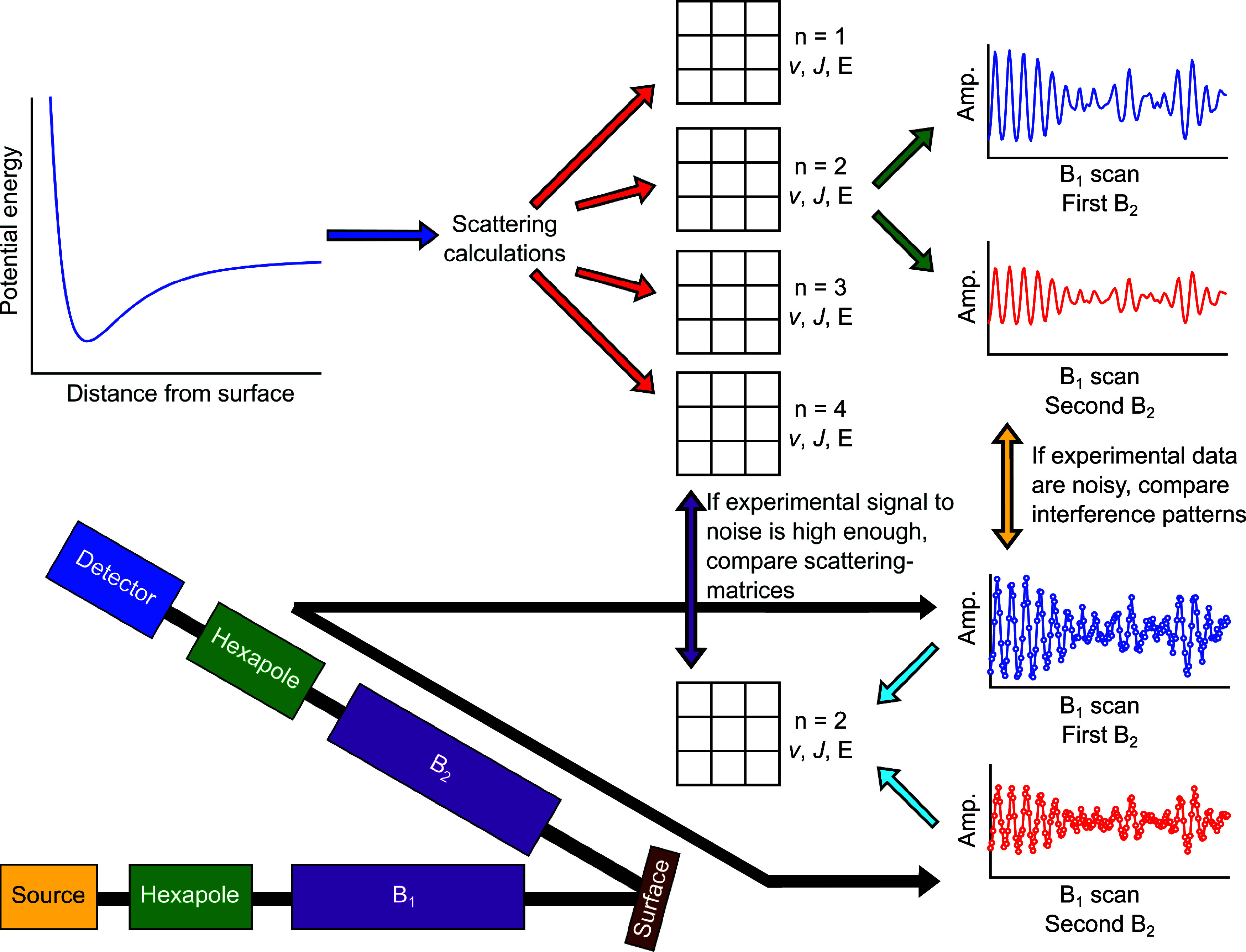
Schematic diagram showing the links between the concepts, the methods, and the results that are presented in the current work.

When quantum mechanical scattering calculations are used in conjunction with a PES, it is possible to calculate the complex amplitudes which define the molecular wave function, from which we obtain the change in both amplitude and relative phase of each component of the wave function^21−23^ when the molecule scatters from the surface (red arrows in Figure 1). These complex amplitudes can be written as the elements of a scattering matrix, S, which converts the wave function before scattering |ψ⟩ to that after scattering, i.e., |ψ′⟩ = S|ψ⟩, Generally speaking, S is a very large matrix, however, when we limit ourselves to a particular incoming and outgoing energy (*E*), a particular vibrational (*v*) and rotational state (*J*) and a particular diffraction channel (*n*), the dimensions of the S-matrix reduce drastically. For example, for the elastic scattering of a *J* = 1 H_2_ molecule, as will be discussed in this paper, the relevant S-matrix is a 3 × 3 matrix (shown schematically in Figure 1 as the 3 × 3 grids for each *n*, *v*, *J* and *E*) consisting of 9 complex numbers linking the *m*_*J*_ components (rotational projection states) of the incoming wave function with the *m*_*J*_ components of the scattered wave function.

## Methods

Over the past few years, we have been developing a magnetic molecular interferometry (MMI) technique which allows us to study H_2_ and D_2_ scattering from surfaces, the details of which have been published previously^24−27^ and are summarized in Section S1 of the Supporting Information (SI). For the purpose of this work, we can use a very schematic description of an MMI experiment depicted in the bottom left of Figure 1, which can be split into three distinct stages. (I) Molecules emitted by the source approach the surface in a superposition of *m*_*J*_ and nuclear spin (*m*_*I*_) projection states which we select and control using a combination of a hexapole lens and a magnetic field B_1_. (II) They scatter from the surface in various directions, where the probability of scattering in a particular direction as well as any changes in amplitude or phase of the superposition state depend on the interaction with the surface, i.e., are dependent on the PES. (III) The superposition state of the scattered molecules is manipulated by a second magnetic field B_2_, before they pass through a second hexapole field and reach the detector, which produces a signal which is a function of the quantum state of the molecule as it entered the hexapole. Measuring this signal for various combinations of the two manipulation fields B_1_ and B_2_ leads to interference patterns which are the observable of the experiment (black arrows in Figure 1).

As in an MMI experiment we usually look at a particular incident energy and scattering geometry, and as the nuclear spin is assumed to be a spectator to the collision event, the scattering can be characterized by only the changes which occur in the *m*_*J*_ elements of the wave function. As mentioned earlier, for the case of ortho-H_2_ in its ground rotational state (*J* = 1), the scattering matrix can be written as a 3 × 3 matrix, the elements of which represent the change in the amplitude and phase for each of the *m*_*J*_ basis states. It is important to note that the same PES will produce different scattering matrices for different translational energies and for different diffraction channels, as is illustrated schematically in Figure 1. On the other-hand each scattering matrix will give rise to different interference patterns measured by the MMI which depend on the B_1_ and B_2_ fields used in the experiment.

The various MMI measurements which have been performed so far have been analyzed and compared to theory using different approaches, reflecting development of the methodology, the availability of PESs, and the type and quality of the experimental data. Below we briefly describe the approaches used so far and the one used in this study.

In the first proof of principle study,^24^ interference patterns (B_1_ scans for a fixed B_2_ value) were measured for flat (111) and stepped (511) copper surfaces. For the stepped surface there was no PES to compare with, leaving the published interference patterns as benchmarks for future theoretical work. For the Cu(111) surface a DFT based PES existed, and a comparison was made using the following scheme which is illustrated schematically in Figure 1. Combining a DFT based PES calculated using the optPBE-vdW exchange correlation functional, and quantum scattering calculations an S-matrix was calculated (blue and red arrows in Figure 1). This S-matrix was then used to simulate an MMI signal (green arrows in Figure 1) using methods described previously^26,28^ and in Section S2 of the SI, which was then compared with the experiments (yellow arrow in Figure 1). The measured and simulated interference patterns differed both in their relative oscillation amplitude and in the pattern itself, indicating that while the PES used was successful in reproducing other experimental observables,^29^ it is not accurate enough to reproduce the subtle properties the MMI setup is sensitive to.

A different, more advanced level of analysis was developed and implemented for analyzing MMI measurements of elastic scattering of H_2_ from LiF(100).^25^ Here, theoretical S-matrices were not available, similarly to the case for the Cu(511) data mentioned earlier, but instead of just publishing the measured interference patterns, the elements of the S-matrix were successfully extracted from the measured data (light blue arrow in Figure 1). The details of the fitting procedure used to perform this have been published previously,^26^ and are summarized in Sections S2 and S3 of the SI. Simply speaking, this was done by calculating an interference pattern from an S-matrix as was done to simulate the Cu(111) data,^24^ but instead of using a DFT based S-matrix, a random matrix was used as a starting point and its elements were optimized until the two calculated interference patterns (B_1_ scans for different B_2_ values) best fitted the corresponding experimental curves. The fitting procedure was shown to be capable of finding a unique set of S-matrix elements from the measured interference patterns. In contrast to the Cu(111) results, the availability of an experimentally determined S-matrix means theoreticians can easily assess any DFT potential they develop in the future without the need to perform detailed simulations of the MMI interference patterns. Furthermore, the fitted scattering matrices allowed us to characterize the polarizing properties of the LiF crystal, i.e., a nonpolarized beam scattered from a LiF crystal becomes polarized, where the stereodynamic preferences are different for the two diffraction peaks studied.

Another more recent example of extracting S-matrix elements from a measurement was a surface temperature dependent study of the interference patterns measured for the specular scattering of H_2_ from Cu(511).^26^ The interference patterns that were obtained changed as a function of surface temperature between 100 and 550 K, with the specular scattering channel becoming more selective for *m*_*J*_ = 0 molecules at higher surface temperatures, and also more strongly polarizing the scattered beam in *m*_*J*_ = 0 at higher surface temperatures. The dependence of the scattering probabilities for the different *m*_*J*_ states of H_2_ on the surface temperature provides another challenge for theoreticians, as the results demonstrate that it will be necessary to include the surface temperature in any future models that are developed for this particular system, in order to reproduce the empirical scattering matrices.

Whist the ability to extract empirical S-matrix elements from MMI measurements is very attractive, it is not always possible. For example in the case of rotationally inelastic diffraction of D_2_ from Cu(111),^27^ the interference pattern that was obtained is comparatively simple compared to those obtained for the elastic scattering of H_2_, and has a relatively small amplitude which makes it rather noisy. As a result, it was not possible to extract a unique scattering matrix from the data following the same procedure mentioned above. Instead, the measured interference pattern was compared to one simulated using a scattering matrix calculated by a combination of using a DFT PES and a quantum scattering calculation (yellow arrow in Figure 1). The comparison showed discrepancies, from which it could be deduced that the DFT based PES, which has previously successfully reproduced reaction probabilities,^29^ underestimates the probability of Δ*m*_*J*_ ≠ 0 events, demonstrating the increased sensitivity of the MMI technique to the underlying PES and its usefulness for testing theoretical modeling.

Finally, in this paper, we report an MMI study of elastic scattering of ortho-H_2_ in its rotational ground state (*J* = 1) which produces a particularly large set of experimental data. The surface we chose was the same stepped Cu(511) surface mentioned earlier, however instead of studying the interference pattern in specular scattering,^26^ we made use of the fact that this highly corrugated crystal allows us to measure interference patterns from seven different diffraction peaks. As we will show, the type of information and the certainty of the values obtained varies from one diffraction peak to another, but in combination they provide a particularly vigorous challenge for theoretical models as all the observations would have to be correctly described by the same PES.

## Results and Discussion

**Figure 2:**
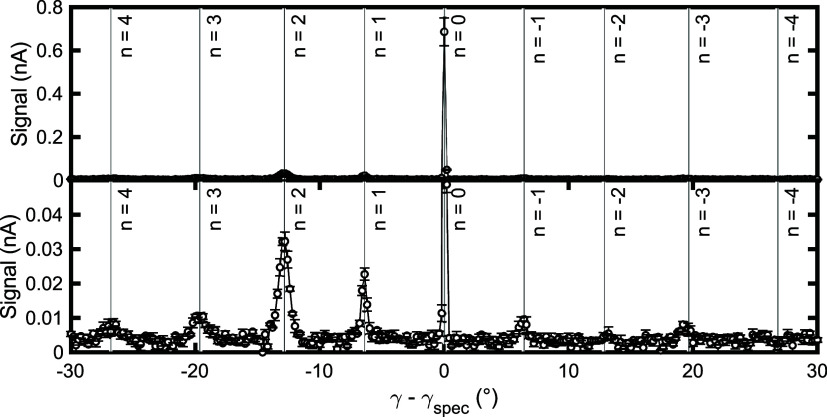
Incident angle scan for H_2_ scattering from a Cu(511) surface showing the positions of the different diffraction peaks measured at a surface temperature of 200 K. The bottom panel is a magnification of the top panel, and the labels show which order of diffraction the peaks correspond to. We note that the asymmetric intensities of the diffraction peaks are expected due to the geometry of the instrument (off-normal incidence angle) as well as the need to reoptimize the tilt angle throughout the incident angle range, as mentioned in the text.

Figure 2 presents an incident angle scan which shows the position of the diffraction peaks for H_2_ scattering from a Cu(511) surface with the azimuth aligned perpendicular to the step edges, with the bottom panel of the figure being a magnification of the top panel. The angle on the *X*-axis corresponds to the position of the peak with respect to the specularly scattered peak. While the specular (*n* = 0) peak is the largest, diffraction peaks up to the third order are visible either side of specular, as well as the *n* = 4 peak. It is important to stress that the continuous scan shown in Figure 2 should not be used to quantitatively compare the relative intensities of the diffraction peaks. Within the large range of angles we measured (60°), the residual misalignment of the crystal leads to a variation of the optimal tilt angle of about 0.5° between the two edges of the measurement. Correspondingly, to measure the height of the peaks requires optimizing the tilt angle for each diffraction peak, a process we performed before the MMI measurements shown below. We also note that the signal intensity when performing MMI measurements at the various diffraction peaks was at least an order of magnitude larger than the diffuse scattering intensity between the diffraction peaks, which includes contributions from both elastic scattering from defects on the surface and from inelastic scattering. Correspondingly, contributions to the MMI signals from any inelastic processes, including phonon creation and annihilation, are expected to be negligible.

**Figure 3:**
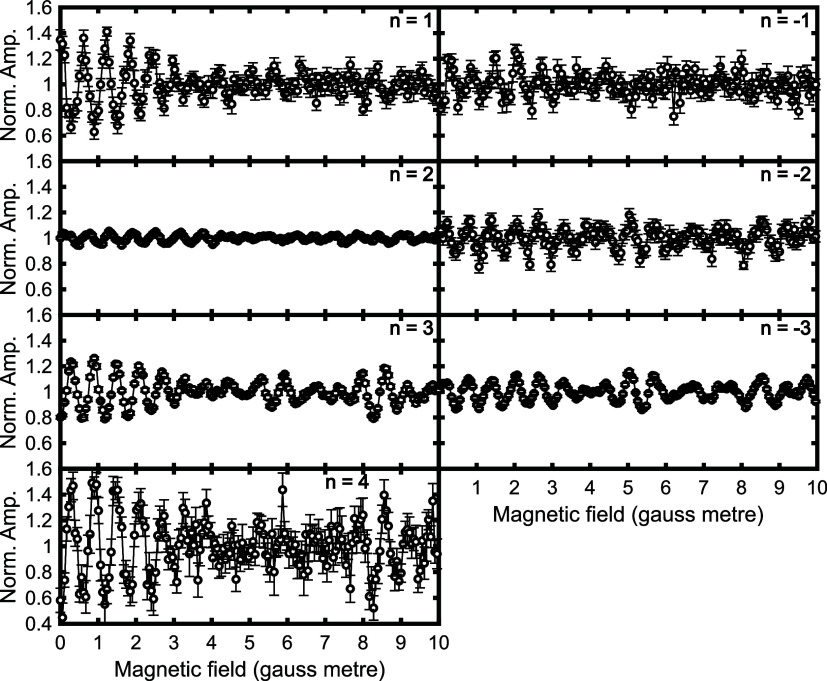
Normalized interference patterns for H_2_ scattering into the various different diffraction channels on a Cu(511) surface at a fixed temperature of 200 K. The magnetic field in the first solenoid was scanned, and that in the second solenoid set to 0 gauss meter.

In the current work, we will focus on the MMI interference patterns measured for the different diffraction peaks (*n* ≠ 0) rather than specular, which has been published previously.^26^ The molecules contributing to the signal in nonspecular diffraction channels have a much narrower velocity spread due to the angular resolution of the machine,^25^ and therefore correspond to a more well-defined collision energy than measurements performed on specular, which make them better for comparison with results from theoretical models. Interference patterns obtained by scanning B_1_ with a fixed field of B_2_ = 0 gauss meter for the different diffraction peaks are presented in Figure 3, where the text in each panel shows which diffraction peak the data corresponds to. All the plots are on the same *Y*-axis scale, which immediately shows that the amplitude of the oscillations is different for the different diffraction peaks, with the *n* = 1 and 4 peaks showing bigger oscillations than the *n* = 2 peak, which is approximately a factor of 10 smaller. The oscillation patterns can also be different, for example the oscillations for the *n* = 1 and 4 peaks are out of phase with each other, although there are also similarities in some of the measurements, for example those for the *n* = −2 and −3 diffraction peaks. All these measurements clearly contain a wealth of information about the rotational orientation dependence of the molecule scattering into the different diffraction peaks and correspondingly the interaction potential that underpins the molecule–surface collision.

For each diffraction peak, a second measurement was performed scanning B_1_ at a value of B_2_ = 11.2 gauss meter, which is needed in order to accurately extract a S-matrix from the experimental data.^25^ The methods used to calculate signals and extract S-matrices from interference measurements have been described in detail previously^26^ and are summarized in Section S3 of the SI, so only the key details are presented here. Note that this corresponds to the light blue arrows in Figure 1. Briefly, two sets of 100 fits are run with randomized initial parameters to ensure that the values we obtain from the fit correspond to the global minimum of the parameter space, and not the local minimum. In the first set of 100 fits, two parameters which account for uncertainties in the hexapole transmission probabilities,^28^ two parameters which characterize the velocity distribution of the beam, a background parameter and the scattering matrix elements are allowed to vary. In the second set of fits, only the scattering matrix parameters and background are allowed to vary, with the other parameters fixed at the best-fit values from the first 100 fits. As the surface azimuth was aligned so that the scattering plane was perpendicular to the step edges, constraints were applied to the scattering matrix elements due to the reflection symmetry,^27^ which in effect reduces the number of free S-matrix values that are being fit to eight.

**Figure 4:**
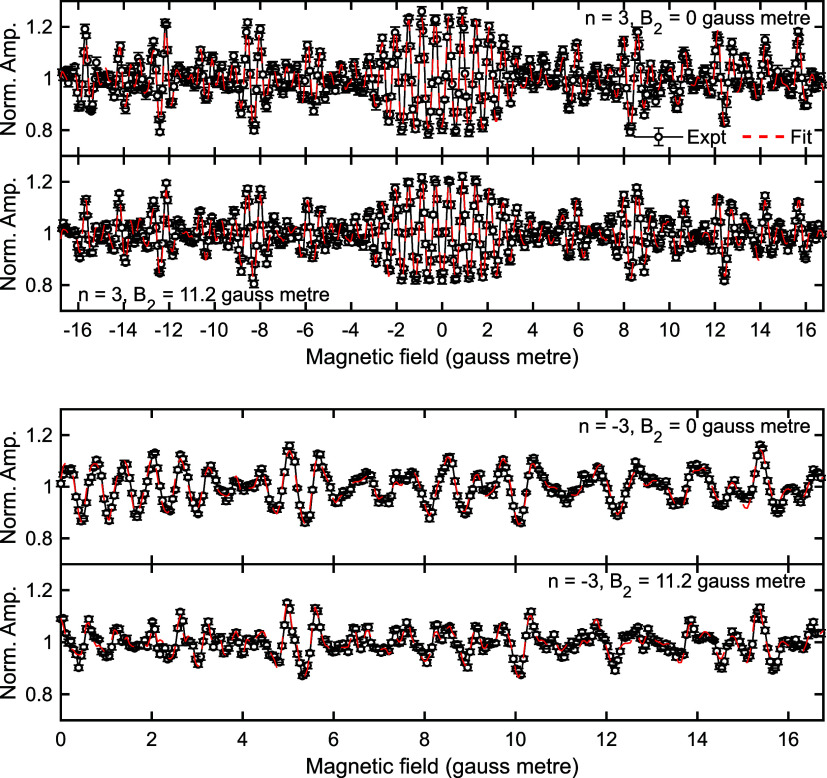
Fits to the MMI interference patterns measured for the *n* = 3 diffraction peak scanning B_1_ at a value of B_2_ = 0 gauss meter (top panel) and B_2_ = 11.2 gauss meter (second panel), and for the *n* = −3 diffraction peak scanning B_1_ at a value of B_2_ = 0 gauss meter (third panel) and B_2_ = 11.2 gauss meter (bottom panel) for H_2_ scattering from Cu(511) at a surface temperature of 200 K.

Our discussion of the results will focus on the results for the *n* = ±3 diffraction peaks first. Fits to the data are shown in Figure 4, with the top two panels presenting the experimental data (black) and fit (red) to the two measurements for the *n* = 3 diffraction peak, and the bottom two the same for the *n* = −3 diffraction peak. The fits follow the complicated oscillation patterns measured in both data sets extremely well, allowing us to obtain a scattering matrix which characterizes the experimental data, which can then be compared with the results from theoretical models. However, the significance of this procedure depends on how accurately we can determine the scattering matrix elements from the fit, as any errors in the parameters would need to be considered in any future comparison with theory-based S-matrices.

As we will show below, the uncertainty in the fitted S-matrix elements depends on both the signal-to-noise of the measurement and on the pattern itself, which in turn depends on the S-matrix. To determine the specific uncertainties for the various signals measured in this study we proceeded as follows. We first ran the regular fitting procedure^26^ to obtain the most likely value for all the elements of the different S-matrices for the different diffraction peaks. Next, for each diffraction peak, we generated two interference patterns (corresponding to the two different values of B_2_) by simulating MMI signals using the most likely S-matrix element values which we obtained from the fit to the corresponding experimental data. By adding random noise to each of these simulated signals, we created ten different repetitions of each of these signals with uncertainties which mimic those in the experimental data. The noise level was chosen to accurately represent the uncertainties in the corresponding experimental data set (specifically, the noise was randomly sampled from a normal distribution that had the same standard deviation as the measured data had which is shown by the error bars in Figure 3). Each of these ten signals was then fitted 100 times in the same way as the experimental data was analyzed. The parameters from the 20 fits with the lowest errors from each of the ten signals were then combined to produce a data set of 200 fits. These fit results allow us to plot histograms as well as calculate the mean value and the standard deviation, all of which allow us to assess the accuracy and reliability of the S-matrix fitting process.

**Figure 5:**
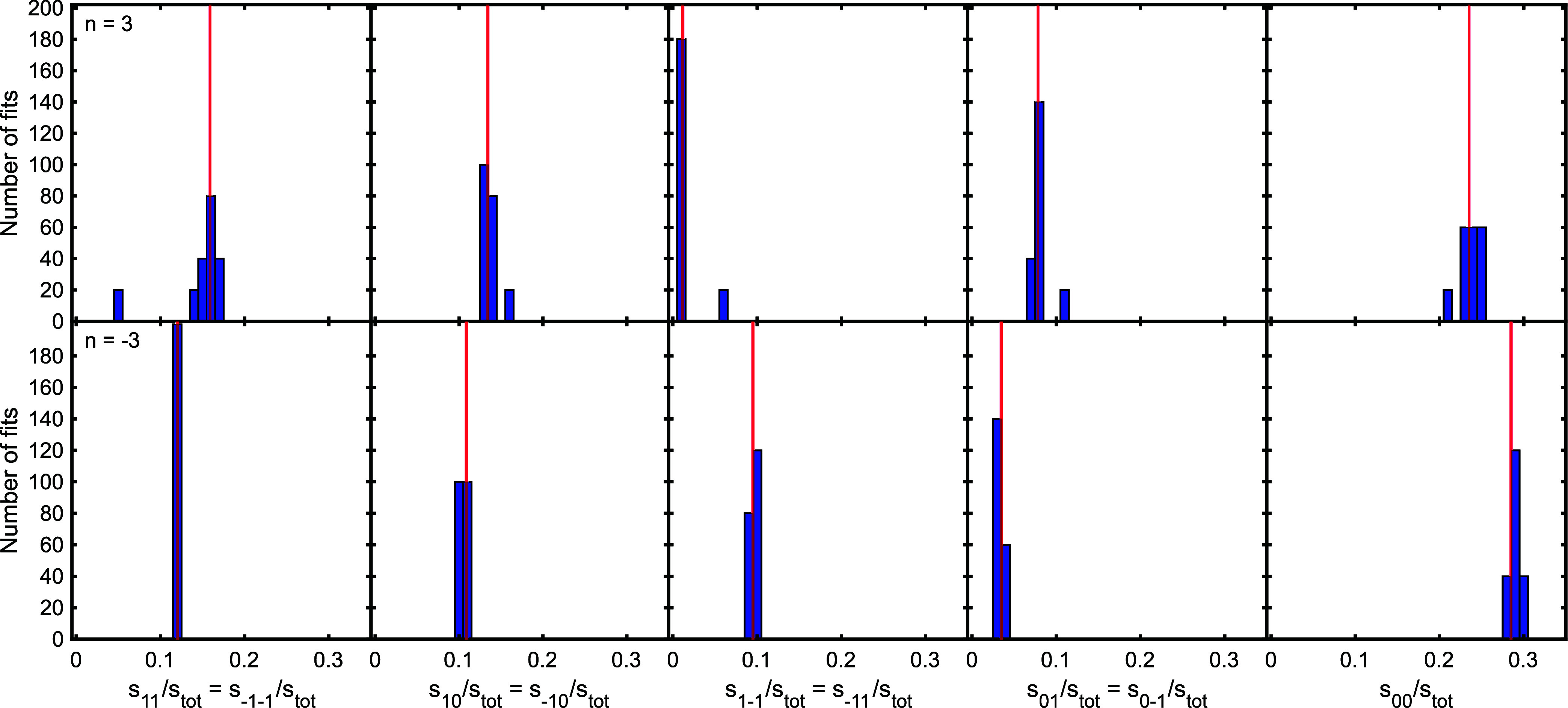
Histograms of scattering matrix amplitudes obtained by taking the best 20 fits with the lowest errors from fitting 10 different simulated signals with noise added which reflects the uncertainty in the experimental data (blue bars). The values which were used to simulate patterns before noise was added are shown by the vertical red lines. See text for details.

Figure 5 presents a histogram of the scattering matrix amplitudes, with the values obtained for the *n* = 3 and −3 diffraction channels in the top and bottom row of panels, respectively. The amplitudes have been normalized to the sum of the amplitudes of all the scattering matrix elements, and the red vertical line in each panel shows the value of the parameter that was used to generate the simulated patterns before the random noise was added to them. The *X*-axis label identifies the element we are plotting the amplitude of, using the notation, s_f’*n*’_, where *n*’ corresponds to the *m*_*J*_ state immediately before the collision, and f’ the *m*_*J*_ state after the collision. In the case of the *n* = −3 data, presented in the bottom row of Figure 5, the amplitudes obtained from the fit show very little spread and are clustered around the value used to simulate the signals that were fit. This means that the empirical S-matrix values obtained from the fits to the experimental data can be treated with a high level of certainty. For the *n* = 3 peak, presented in the top row of Figure 5, there is more spread in the values of the parameters that are obtained. While the results from fits of 9 out of the 10 simulated signals cluster around the value that was used to simulate the signals that were then fit to, there is one outlier corresponding to the 20 fits obtained when fitting the 10th signal which appears in all panels in the top row away from the main cluster, showing that there is a larger uncertainty in the parameters obtained for the *n* = 3 diffraction peak. These results demonstrate that while a reliable scattering matrix can be obtained for one of these two diffraction peaks, there is more uncertainty in the values of the other scattering matrix, even though the noise levels (error bars) for both sets of MMI measurements are comparatively small.

**Figure 6:**
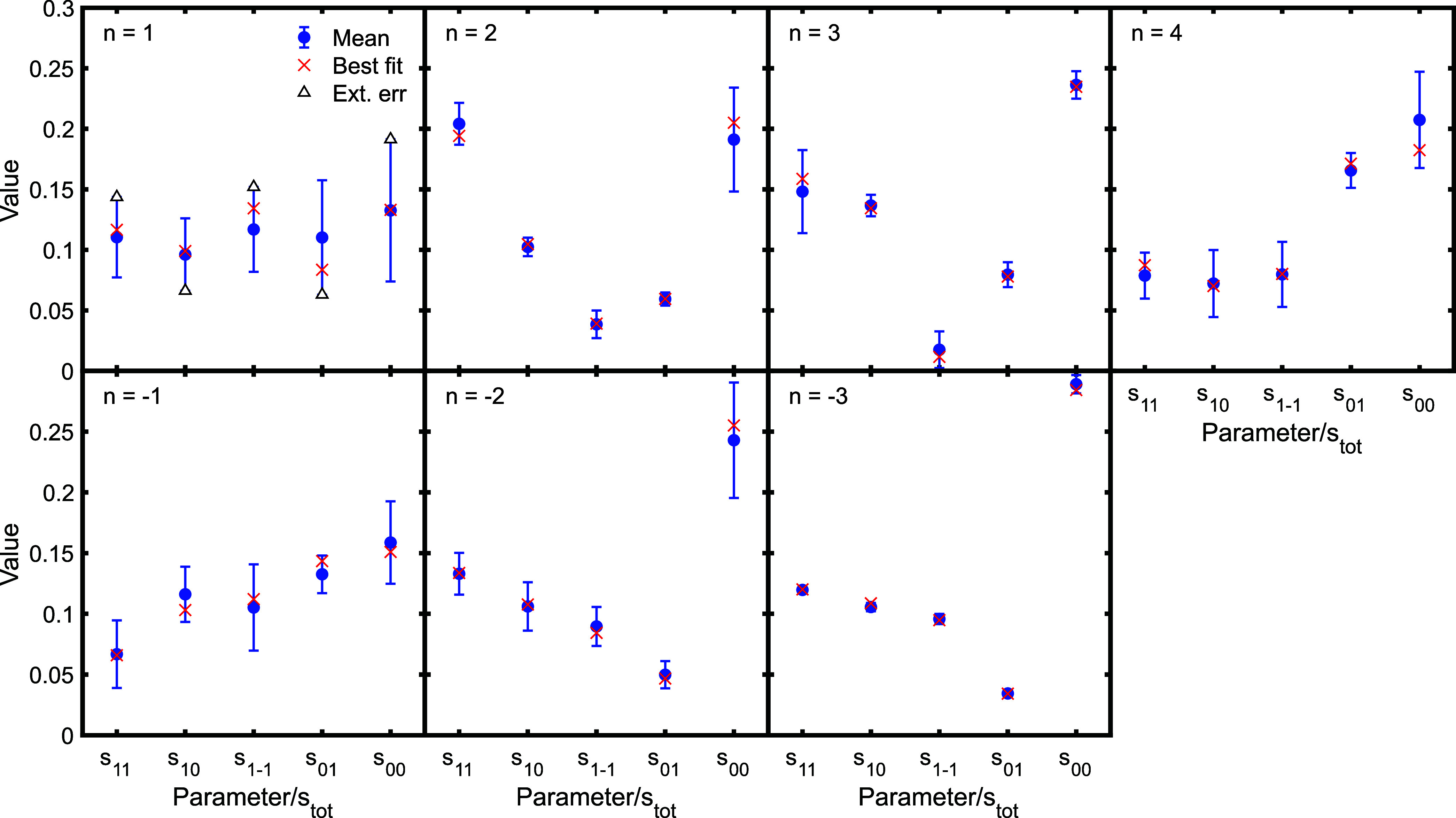
Unique scattering matrix amplitudes normalized with respect to the sum of the scattering matrix amplitudes obtained by averaging the results of the best 20 fits with the lowest errors from each of fits to 10 different simulated signals with noise which reflected the noise level in the experimental data (blue circles) alongside the value obtained from the best fit to the experimental data (red crosses). The additional points in the first panel (triangles) correspond to values lying at the extreme of the error bars. See text for details.

**Figure 7:**
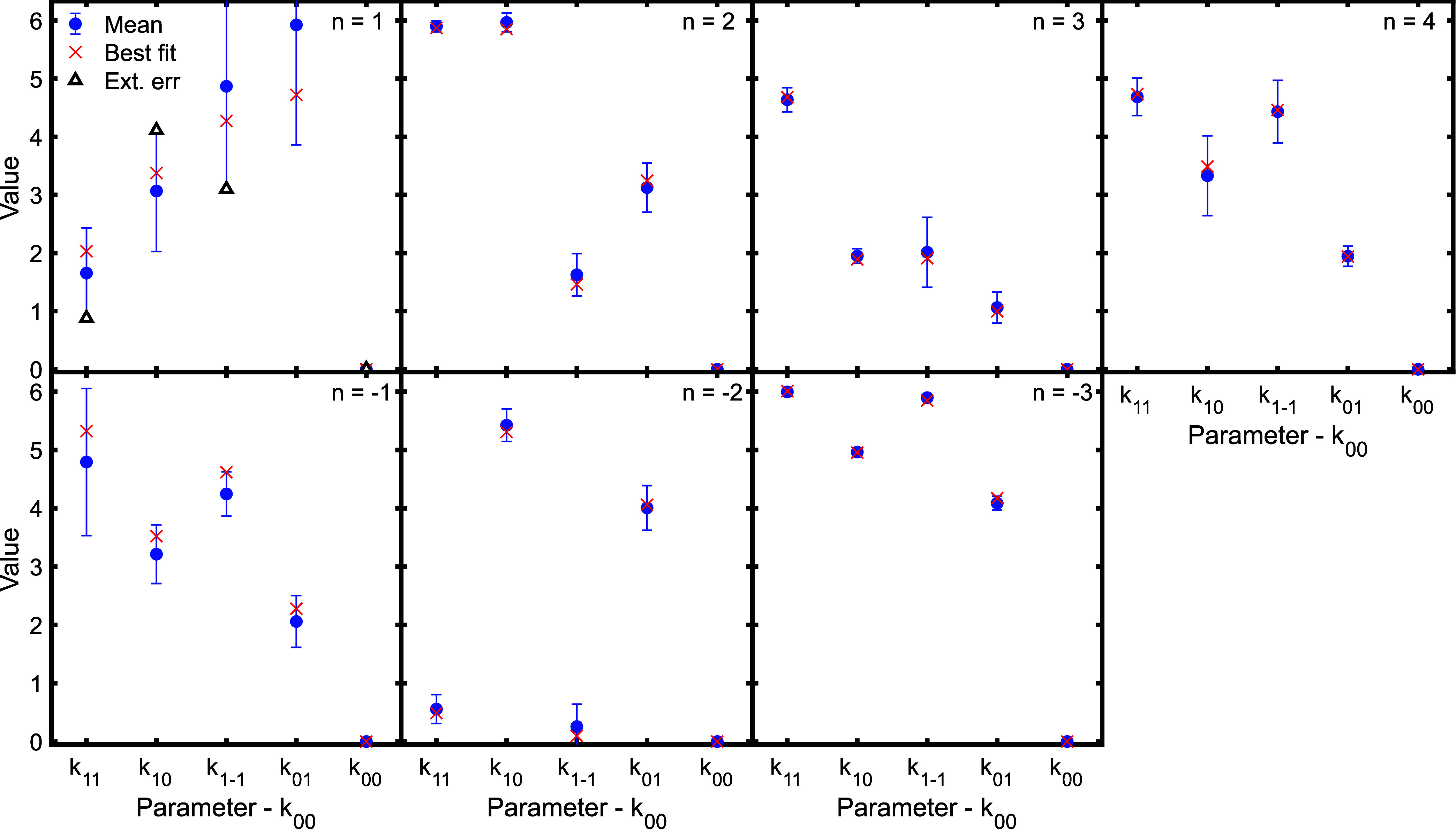
Unique scattering matrix phases with respect to *k*_00_ obtained by averaging the results of the best 20 fits with the lowest errors from each of fits to 10 different simulated signals with noise which reflected the noise level in the experimental data (blue circles) alongside the value obtained from the best fit to the experimental data (red crosses). The additional points in the first panel (triangles) correspond to values lying at the extreme of the error bars. See text for details.

For the other diffraction peaks, the uncertainties in the experimental data tend to be larger than those for the *n* = ±3 channels, as demonstrated by the larger error bars in Figure 3. We followed the same procedure described above to assess the reliability of the scattering matrix elements extracted from these measurements. The results for the scattering matrix amplitudes are presented in Figure 6, and the phases in Figure 7, with the text in the panel stating which diffraction peak each panel corresponds to. Here, the results are presented as the mean scattering matrix parameter (blue circle) with an error bar that reflects the spread (standard deviation) in the values obtained, with the amplitudes given with respect to the sum of the S-matrix amplitudes for that channel, and the phases (*k*_f’*n*’_) with respect to the *k*_00_ parameter. The red crosses mark the values which were obtained when fitting the experimental data and were then used to generate the simulated data before noise was added (this is equivalent to the red vertical lines in Figure 5, and referred to below as the “true value”). These results are also presented in Table S1 of the SI, which gives the value of the parameters obtained from fitting the experimental data alongside the mean value and uncertainty obtained from analyzing the simulated signals.

Looking at the results in Figure 6, we see that for some diffraction peaks our results determine all the individual S-matrix amplitudes quite uniquely and are very close to the true value (for example, the *n* = −3 diffraction peak in the third panel of the bottom row). For others, some of the matrix elements are well-defined and others less so (for example, the *n* = 2 diffraction peak in the second panel of the top row), and for some measurements, the uncertainty makes comparisons between the S-matrix elements meaningless (for example the *n* = 1 diffraction peak in the first panel of the top row). The conclusions that can be drawn in each case therefore vary depending on the uncertainties in the parameters that are obtained.

An important point to stress is that while it is convenient to present the amplitudes and phases of the scattering matrix elements using their mean value and standard deviation, if we calculate a signal using the mean value of all the scattering matrix parameters it will not necessarily produce an interference pattern that will match the experimental data. This is because the signal is an interference measurement which depends sensitively on the amplitude and phase of each component. Correspondingly, there is a correlation between the value of one matrix element and all other elements, and while you can fit the data quite well using any value within its own distribution, this choice will only work if the other elements are changed in a certain way within their own distributions. This can be visualized by noting that the parameters of the best fit to the experimental data (red crosses in Figures 6 and 7) do not necessarily follow the mean value (blue circles at the center of the error bars).

**Figure 8:**
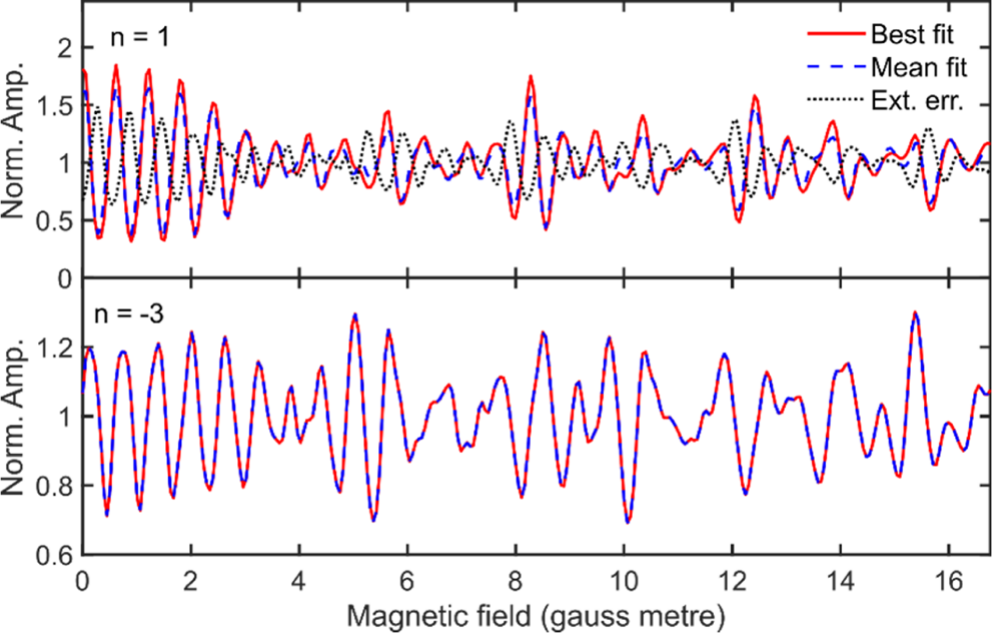
Comparison of the simulated MMI signals for the *n* = 1 (top panel) and *n* = −3 (bottom panel) diffraction channels produced using the scattering matrix parameters obtained from the best fits to the experimental data (shown as red crosses in Figures 6 and 7) and the mean of the best fits obtained from fitting simulated data with similar noise levels to the experimental data (shown as blue circles in Figures 6 and 7). For the *n* = 1 channel, an additional simulated signal is shown (black dotted line) corresponding the points lying at the extremes of the error bars of the parameters (shown as triangles in the top left panels of Figures 6 and 7). See text for details.

Figure 8 further demonstrates this rather subtle point, showing a comparison of signals calculated using the best fit scattering matrix elements (red line) and those obtained using the mean parameters (blue dashed line) for the *n* = 1 (top panel) and *n* = −3 (bottom panel) diffraction peaks. For the *n* = −3 channel, the two signals are indistinguishable. In the case of the *n* = 1 channel, there are small differences between the two signals, reflecting the larger uncertainties in the extracted scattering matrix values, although they are still quite similar. If the mean and best fit parameters were more significantly different, this would have a larger effect on the two signals. As an extreme example of this, consider a case where the best fit parameters correspond to the values at the alternating extremes of the error bars for the *n* = 1 peak, which are shown as triangles in the top left panels of Figures 6 and 7. The signal calculated for these values of the S-matrix parameters is shown as a black dotted line in the top panel of Figure 8. Comparing the signal obtained using the mean value and this extreme case, it can clearly be seen that these two interference patterns are different, demonstrating that not any choice of parameters within the distribution width will be able to reproduce the experimental signal.

Returning our attention to Figure 6, the smallest uncertainties are seen for the *n* = −3 diffraction channel (third panel, bottom row), with the scattering matrix parameters for the *n* = 2 and 3 channels also having comparatively small uncertainties (second and third panels, top row). As the error bars on these scattering matrix parameters are comparatively small, the scattering matrix elements for these diffraction channels could be compared directly with the results of theoretical calculations, as shown by the purple arrow in Figure 1. It also allows us to extract information about the *m*_*J*_ state to *m*_*J*_ state scattering probabilities, which correspond to the square of the scattering matrix amplitudes, for example the results for the *n* = 2 diffraction peak (second panel, top row) show that Δ*m*_*J*_ = 0 collisions are more likely than those that change *m*_*J*_ state (i.e., the diagonal s_11_ and s_00_ parameters shown as the first and fifth point in the panel are larger than the off-diagonal s_10_, s_01_ and s_1–1_ parameters shown by the three points in between). While the uncertainties are larger for the parameters that are obtained for the *n* = −2 diffraction peak (second panel, bottom row), which makes it more difficult to draw quantitative conclusions from the data, the trend in the scattering matrix elements mirrors those seen for the *n* = −3 diffraction peaks (third panel, bottom row), reflecting the similarity of the oscillation patterns that were measured for these two channels (right-hand column, Figure 3).

The uncertainties in the scattering matrix elements obtained from the data for the *n* = 4 diffraction peak (fourth panel, top row) are larger. Nevertheless, the results still show that the molecules scattered into that channel are more likely to be rotating like a cartwheel than a helicopter (as the s_0*n*’_ parameters shown by the last two points in the panel are consistently larger than the s_1*n*’_ parameters shown by the first three points in the panel, with the differences being larger than the size of the error bars). This trend could serve as a comparison point with a theoretical calculation. In contrast the trends in the scattering matrix elements before collision (comparing s_f’1_, s_f’0_ and s_f–1_, i.e., the first three points in the panel) are less clear-cut as the values obtained for all three points all fall within the uncertainties of the parameters. Therefore, for this diffraction peak, the comparison between experiment and theory would be a combination of comparing S-matrix elements (i.e., the purple arrow in Figure 1) and the interference signals that are obtained (i.e., the yellow arrow in Figure 1).

The uncertainty of the empirical scattering matrix parameters for the *n* = ±1 diffraction peaks (first panel in the top and bottom rows of Figures 6 and 7) means that less can be said about the scattering process and the values that the parameters take for these channels, as nearly all the values that are obtained fall within the error bars of the other parameters, i.e., the uncertainty on each parameter is larger than any differences between them. However, this does not mean that these measurements have no value. While they cannot provide a definitive scattering matrix, the interference patterns can be compared to those calculated using S-matrices obtained from theoretical methods (corresponding to the yellow arrow in Figure 1). If the calculated interference pattern does not match the experimentally measured one, then this still shows that there is an inaccuracy in the calculation. If, on the other hand, the calculated curve does reproduce that measured experimentally, then this provides evidence that the calculations could be accurate. In the case of the current work though, this would be a necessary condition but not a sufficient condition, as there are scattering matrix elements and/or interference patterns for a number of other diffraction peaks that the calculations would also have to reproduce. Taken together, these results provide a stringent benchmark for gas–surface scattering calculations and demonstrate the wealth of information that can be obtained using the MMI technique when studying scattering from a corrugated surface.

## Conclusions and Outlook

The current work presents a study focusing on the rotational orientation dependence of the multiple diffraction channels that are observed for H_2_ scattering from a Cu(511) surface. Interference patterns have been presented for seven different diffraction peaks, which qualitatively show the differences or similarities between the rotational orientation dependence of the scattering for the different diffraction channels. In the cases where the noise on the measurements is small, it has been demonstrated that it is possible to extract the scattering matrix elements with low levels of uncertainty and provide insight into the *m*_*J*_ state to *m*_*J*_ state scattering probabilities. For the measurements where the errors on the measurements are larger, it is not possible to make such quantitative comparisons, although certain trends can still be identified in some cases. While these diffraction channels arguably provide less information about the underlying PES than those measured with lower errors, they still have value in the sense that any theoretical model must produce a scattering matrix which when used to calculate an MMI signal reproduces the interference pattern observed experimentally. In combination, these results, along with the results for the other diffraction channels, and the specular measurements published previously,^24,26^ provide several benchmarks that any future calculation should strive to reproduce. While it might be possible for a model to fortuitously reproduce one or two of the experimental results and still not be quite right, this is significantly less likely if the model can accurately reproduce all of the data now available for H_2_ scattering from a Cu(511) surface.

Finally, to fully test theoretical models, it could be that the measured data which is now available is still not sufficient. It may also be necessary to measure the rotational orientation dependence at a different incident energy of the beam, which would mean the collision would probe different regions of the potential energy surface. Likewise, studying rotationally or translationally inelastic scattering would also provide complementary information about the underlying interaction potential.

## Supporting Information Available

Additional details about the experimental, signal calculation, and data analysis methods used in the current study (PDF)
