# Supplementary material for: Empirical Determination of Scattering Matrices from Magnetic Molecular Interferometry for Gas–Surface Collisions
Source: J Phys Chem C Nanomater Interfaces. 2024 Dec 2;128(49):20913–22. doi: 10.1021/acs.jpcc.4c06913 (PMC11648075; doi:10.1021/acs.jpcc.4c06913)
Supplement: Supplementary file 1 — jp4c06913_si_001.pdf [file jp4c06913_si_001.pdf]

Supporting Information for Publication

Empirical Determination of Scattering-Matrices from Magnetic Molecular Interferometry for Gas-Surface Collisions

Helen Chadwick and Gil Alexandrowicz

Department of Chemistry, Faculty of Science and Engineering, Swansea University, Swansea, SA2 8PP, UK

Corresponding authors:
h.j.chadwick@swansea.ac.uk g.n.alexandrowicz@swansea.ac.uk

S1. Experimental methods

The experimental apparatus used in the current study is the same as that used previously[1–9], and so only a summary will be presented here.

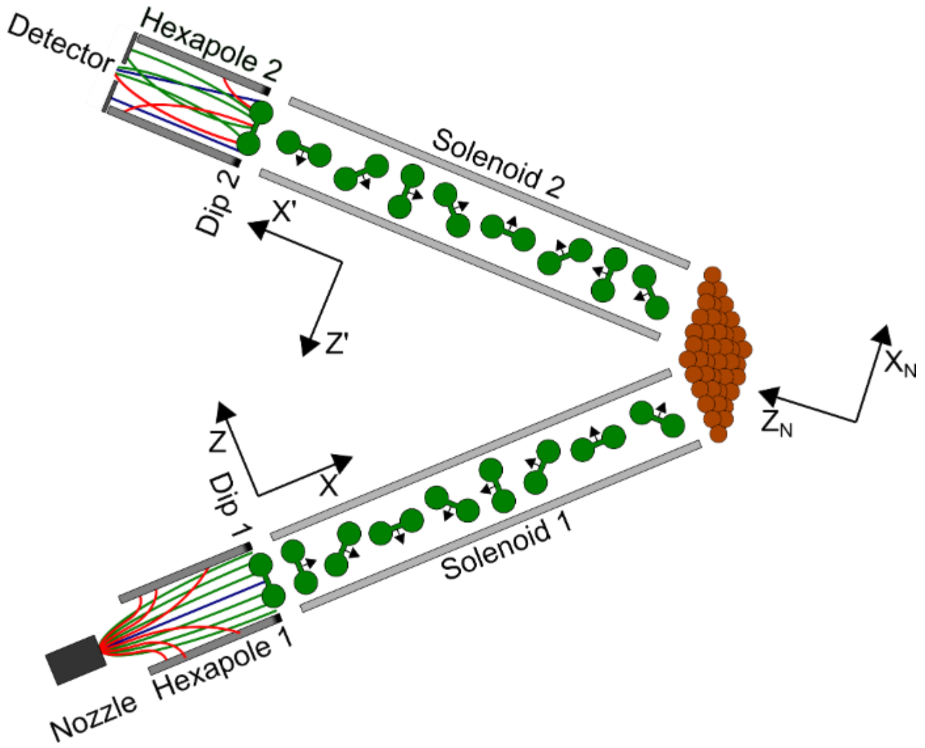

Figure S1. Overview of the magnetic molecular interferometer (MMI) apparatus, showing the positions of the different magnetic elements and the reference frames that will be used in the text.

In brief, the beamline, which is shown schematically in Fig. S1, consists of inhomogeneous and homogeneous magnetic fields which deflect and coherently control the 9 m_I, m_J, states of ortho-H_2 in the state I = 1, J = 1, both before and after the molecule collides with a Cu(511) surface. At the start, a supersonic expansion from a nozzle held at 100K is used to generate the molecular beam of H_2, which gives a velocity on the order of 1450ms^{-1}. This enters a magnetic hexapole[10], where the different m_I, m_J states are deflected depending on their magnetic moment. Within the strong magnetic field gradients, the superposition states decohere[11] meaning at the end of the first hexapole the beam consists of 9 unequally populated, but pure, m_I, m_J states. After a zero-field region, the molecules enter the first solenoid controlled by a high stability Danfysik power supply calibrated to control currents of 0 – 10A with ppm accuracy. The solenoid generates a magnetic field along the X direction, where the superposition of the different m_I, m_J states undergo Rabi oscillations, allowing the coherent control of the wave-function of the H_2 molecules that then collide with the Cu(511) surface. The sample (Surface Preparation Labs, Zandaam) is mounted on a 6-axis manipulator in an ultra-high vacuum chamber (base pressure 1 x 10^{-10}mbar) at the end of the first arm of the beamline. This was cleaned by repeated cycles of Ar^+ sputtering performed at 300K and annealing to approx. 700K, with the surface temperature monitored using a T-type thermocouple. The cleanliness and order of the surface was verified by monitoring the signal for a specularly scattered beam and ensuring further cleaning cycles did not improve the signal obtained.

The incident angle between the molecular beam and the surface was changed to allow molecules scattering into different diffraction channels to be directed down the second arm of the beamline. This consists of a second solenoid (controlled by a second, independent Danfysik power supply) which generates a tuneable magnetic field along the X' direction, followed by a second analyser hexapole[12]. At the end of the beamline, the molecules reach the detector, which is a custom built, high sensitivity mass spectrometer detector[13].

S2. Signal calculation

The methods used for analysing the data have also been described previously[1,3,6,9] and so only the main details will be presented here.

To be able to analyse the data, the effect of the different magnetic fields in the apparatus on the 9 different m_I, m_J states of the I = 1, J = 1 molecules needs to be modelled. The probabilities that the different m_I, m_J states are transmitted through the two hexapoles are found using semi-classical trajectory calculations[14]. As the probability that a given state is transmitted through the second hexapole is dependent on its state in the first hexapole[9,15], 81 hexapole probabilities are obtained ($P_{hex}(f,n)$), one for each initial (n) state, final (f) state combination. The coherent evolution of each of the 9 m_I, m_J states through the magnetic fields in the remainder of the apparatus, between the end of the dipole and surface in the first arm, and from the surface to the dipole in the second arm, is calculated semi-classically[7], with the motion of the centre of mass propagated classically and the evolution of the m_I, m_J states calculated using the known magnetic field dependence of the eigenenergies[16]. This produces two propagation matrices, $U(B_1)$ and $U(B_2)$ which characterise the coherent superposition states the initially pure m_I, m_J states become after propagation through the first and second arm of the apparatus respectively.

The wave-function for a molecule at the start of the second hexapole (ψ_n) which was in an initial state $|n\rangle$ at the start of the beamline can be written as

$$|\psi_n\rangle = U(B_2)R(\theta_2)SR(\theta_1)U(B_1)\,|n\rangle \qquad (1)$$

where S is the scattering-matrix. As the surface which is being studied is non-magnetic, m_I is assumed to be a spectator to the collision, meaning that S is a 3 x 3 matrix. Whilst in principle this means that it is parameterised by nine amplitudes and nine phases, due to the reflection symmetry of the scattering event, only five of the amplitudes and five of the phases are unique[3]. Following previous conventions[17], the quantisation axis is taken to be the surface normal, and therefore $R(\theta_1)$ is a rotation matrix which changes the quantisation axis from Z to the surface normal Z_N, and $R(\theta_2)$ a rotation matrix which changes the quantisation axis from Z_N to Z', which is the quantisation axis along which the analyser hexapole selects the states passing into the detector.

The signal at the detector (sig) is obtained by projecting the wave-function given in Eq. (1) onto a final m_I, m_J state f, and then taking the square modulus and summing over the velocity distribution (P_v), which is modelled as a gaussian, and all the initial and final states weighted with the appropriate hexapole transmission probabilities, i.e.,

$$\text{sig} \propto \sum_v P_v \sum_f \sum_n P_{hex}(f,n)\langle f|\psi_n\rangle^2 \qquad (2)$$

S3. Extracting empirical scattering-matrices

The scattering-matrices were obtained by fitting the experimental data using Eqs. (1) and (2) by minimising the difference between the calculated and experimental signals. To achieve this, the Nelder and Mead downhill simplex algorithm[18] was used with simulated annealing to ensure the fits reached the global minimum of the parameter space, as opposed to becoming trapped in a local minimum. Two rounds of 100 fits were used, each with random initial values for the parameters that were allowed to vary in the fit. In the first round of 100 fits, two parameters which account for the uncertainties in the hexapole probabilities[9], the peak and full width at half maximum (FWHM) of the velocity distribution, a background parameter and the 10 unique scattering-matrix elements[6,9] were allowed to vary. In the second round, the parameters for the hexapole probabilities and velocity distributions were fixed at the values which gave the fits with the minimum error in the first round of fitting, and only the background and scattering-matrix elements were allowed to vary. Whilst 10 scattering-matrix elements are allowed to vary in the fit, the normalisation of the amplitudes to the sum of the amplitudes of all the elements, and the phases to k_{00} in effect reduces the number of free S-matrix parameters to 8.

**Scattering matrix parameter**	**Value obtained from fitting experimental data (red cross in Figs. 6 and 7)**				**Mean value obtained from fitting simulated signals (blue circle in Figs. 6 and 7)**				**Estimated uncertainty (error bars in Figs. 6 and 7)**			
**Diffraction peak**	**1**	**2**	**3**	**4**	**1**	**2**	**3**	**4**	**1**	**2**	**3**	**4**
$s_{11}/s_{tot} = s_{-1-1}/s_{tot}$	0.12	0.19	0.16	0.09	0.11	0.20	0.15	0.08	0.03	0.02	0.03	0.02
$s_{10}/s_{tot} = s_{-10}/s_{tot}$	0.10	0.10	0.13	0.07	0.10	0.10	0.14	0.07	0.03	0.01	0.01	0.03
$s_{1-1}/s_{tot} = s_{-11}/s_{tot}$	0.13	0.04	0.01	0.08	0.12	0.04	0.02	0.08	0.04	0.01	0.02	0.03
$s_{01}/s_{tot} = s_{0-1}/s_{tot}$	0.08	0.06	0.08	0.17	0.11	0.06	0.08	0.17	0.05	0.01	0.01	0.01
s_{00}/s_{tot}	0.13	0.21	0.23	0.18	0.13	0.19	0.24	0.21	0.06	0.04	0.01	0.04
$k_{11} - k_{00} = k_{-1-1} - k_{00}$	2.03	5.87	4.68	4.74	1.65	5.90	4.64	4.69	0.77	0.10	0.21	0.32
$k_{10} - k_{00} = k_{-10} - k_{00} + \pi$	3.38	5.85	1.89	3.49	3.07	5.97	1.95	3.33	1.04	0.16	0.13	0.69
$k_{1-1} - k_{00} = k_{-11} - k_{00}$	4.27	1.46	1.91	4.46	4.87	1.63	2.01	4.43	1.77	0.37	0.60	0.54
$k_{01} - k_{00} = k_{0-1} - k_{00} + \pi$	4.72	3.24	1.00	1.94	5.93	3.13	1.06	1.95	2.06	0.42	0.27	0.17
$k_{00} - k_{00}$	0.00	0.00	0.00	0.00	0.00	0.00	0.00	0.00	0.00	0.00	0.00	0.00
**Diffraction peak**	**-1**	**-2**	**-3**		**-1**	**-2**	**-3**		**-1**	**-2**	**-3**	
$s_{11}/s_{tot} = s_{-1-1}/s_{tot}$	0.07	0.13	0.12		0.07	0.13	0.12		0.03	0.02	0.00	
$s_{10}/s_{tot} = s_{-10}/s_{tot}$	0.10	0.11	0.11		0.12	0.11	0.11		0.02	0.02	0.00	
$s_{1-1}/s_{tot} = s_{-11}/s_{tot}$	0.11	0.08	0.09		0.11	0.09	0.10		0.04	0.02	0.00	
$s_{01}/s_{tot} = s_{0-1}/s_{tot}$	0.14	0.05	0.03		0.13	0.05	0.03		0.02	0.01	0.00	
s_{00}/s_{tot}	0.15	0.26	0.28		0.16	0.24	0.29		0.03	0.05	0.01	
$k_{11} - k_{00} = k_{-1-1} - k_{00}$	5.32	0.49	6.01		4.79	0.56	6.00		1.26	0.25	0.03	
$k_{10} - k_{00} = k_{-10} - k_{00} + \pi$	3.52	5.31	4.95		3.21	5.42	4.96		0.50	0.28	0.06	
$k_{1-1} - k_{00} = k_{-11} - k_{00}$	4.62	0.10	5.85		4.25	0.26	5.90		0.38	0.39	0.05	
$k_{01} - k_{00} = k_{0-1} - k_{00} + \pi$	2.28	4.06	4.17		2.06	4.01	4.09		0.44	0.38	0.12	
$k_{00} - k_{00}$	0.00	0.00	0.00		0.00	0.00	0.00		0.00	0.00	0.00	

Table S1. Values of the best-fit and mean scattering-matrix parameters and the associated uncertainties presented in Figs. 6 and 7 of the main manuscript.

References

(1) Alkoby, Y.; Chadwick, H.; Godsi, O.; Labiad, H.; Bergin, M.; Cantin, J. T.; Litvin, I.; Maniv, T.; Alexandrowicz, G. Setting Benchmarks for Modelling Gas–Surface Interactions Using Coherent Control of Rotational Orientation States. *Nat. Commun.* **2020**, *11*, 3110. https://doi.org/https://doi.org/10.1038/s41467-020-16930-1.

(2) Chadwick, H.; Cantin, J. T.; Alkoby, Y.; Alexandrowicz, G. Multiple Echoes in Beam Spin-Echo Spectroscopy and Their Effect on Measurements of Ultra-Fast Dynamics. *J. Phys. Condens. Matter* **2022**, *34* (34), 345901. https://doi.org/10.1088/1361-648X/ac7765.

(3) Chadwick, H.; Somers, M. F.; Stewart, A. C.; Alkoby, Y.; Carter, T. J. D.; Butkovicova, D.; Alexandrowicz, G. Stopping Molecular Rotation Using Coherent Ultra-Low-Energy Magnetic Manipulations. *Nat. Commun.* **2022**, *13*, 2287. https://doi.org/https://doi.org/10.1038/s41467-022-29830-3.

(4) Chadwick, H.; Alexandrowicz, G. Measuring Surface Phonons Using Molecular Spin-Echo. *Phys. Chem. Chem. Phys.* **2022**, *24*, 14198. https://doi.org/10.1039/D2CP01372J.

(5) Chadwick, H.; Alkoby, Y.; Cantin, J. T.; Lindebaum, D.; Godsi, O.; Maniv, T.; Alexandrowicz, G. Molecular Spin Echoes; Multiple Magnetic Coherences in Molecule Surface Scattering Experiments. *Phys. Chem. Chem. Phys.* **2021**, *23* (13), 7673–7681. https://doi.org/10.1039/D0CP05399F.

(6) Chadwick, H.; Alexandrowicz, G. Temperature Dependent Stereodynamics in Surface Scattering Measured through Subtle Changes in the Molecular Wave Function. *Faraday Discuss.* **2024**, *251*, 76–91. https://doi.org/10.1039/D4FD00007B.

(7) Godsi, O.; Corem, G.; Alkoby, Y.; Cantin, J. T.; Krems, R. V; Somers, M. F.; Meyer, J.; Kroes, G. J.; Maniv, T.; Alexandrowicz, G. A General Method for Controlling and Resolving Rotational Orientation of Molecules in Molecule-Surface Collisions. *Nat. Commun.* **2017**, *8*, 15357. https://doi.org/https://doi.org/10.1038/ncomms15357.

(8) Litvin, I.; Alkoby, Y.; Godsi, O.; Alexandrowicz, G.; Maniv, T. Parallel and Anti-Parallel Echoes in Beam Spin Echo Experiments. *Results Phys.* **2019**, *12*, 381–391. https://doi.org/https://doi.org/10.1016/j.rinp.2018.09.032.

(9) Chadwick, H. Characterisation of Magnetic Atomic and Molecular Beamlines for the Extraction of Empirical Scattering-Matrices. *Phys. Chem. Chem. Phys.* **2024**, *26*, 19630. https://doi.org/10.1039/D4CP01785D.

(10) Jardine, A. P.; Fouquet, P.; Ellis, J.; Allison, W. Hexapole Magnet System for Thermal Energy ^{3}He Atom Manipulation. *Rev. Sci. Instrum.* **2001**, *72* (10), 3834–3841. https://doi.org/https://doi.org/10.1063/1.1405794.

(11) Utz, M.; Levitt, M. H.; Cooper, N.; Ulbricht, H. Visualisation of Quantum Evolution in the Stern–Gerlach and Rabi Experiments. *Phys. Chem. Chem. Phys.* **2015**, *17* (5), 3867–3872. https://doi.org/10.1039/C4CP05606J.

(12) Dworski, S.; Alexandrowicz, G.; Fouquet, P.; Jardine, A. P.; Allison, W.; Ellis, J. Low Aberration Permanent Hexapole Magnet for Atom and Molecular Beam Research. *Rev. Sci. Instrum.* **2004**, *75* (6), 1963–1970.

https://doi.org/10.1063/1.1753093.

(13) Bergin, M.; Ward, D. J.; Lambrick, S. M.; von Jeinsen, N. A.; Holst, B.; Ellis, J.; Jardine, A. P.; Allison, W. Low-Energy Electron Ionization Mass Spectrometer for Efficient Detection of Low Mass Species. *Rev. Sci. Instrum.* **2021**, *92* (7), 73305. https://doi.org/10.1063/5.0050292.

(14) Krüger, C.; Lisitsin-Baranovsky, E.; Ofer, O.; Turgeon, P. A.; Vermette, J.; Ayotte, P.; Alexandrowicz, G. A Magnetically Focused Molecular Beam Source for Deposition of Spin-Polarised Molecular Surface Layers. *J. Chem. Phys.* **2018**, *149* (16), 164201. https://doi.org/10.1063/1.5048521.

(15) Alkoby, Y. Studying Molecule-Surface Interactions Using Magnetically Manipulated Molecular Beams, Swansea University, 2022. https://doi.org/10.23889/SUthesis.62110.

(16) Ramsey, N. F. Theory of Molecular Hydrogen and Deuterium in Magnetic Fields. *Phys. Rev.* **1952**, *85* (1), 60–65. https://doi.org/10.1103/PhysRev.85.60.

(17) Mowrey, R. C.; Kroes, G. J. Application of an Efficient Asymptotic Analysis Method to Molecule–Surface Scattering. *J. Chem. Phys.* **1995**, *103* (3), 1216–1225. https://doi.org/10.1063/1.469831.

(18) Nelder, J. A.; Mead, R. A Simplex Method for Function Minimization. *Comput. J.* **1965**, *7* (4), 308–313. https://doi.org/10.1093/comjnl/7.4.308.
